# Application of Non-Blood-Derived Fluid Biopsy in Monitoring Minimal Residual Diseases of Lung Cancer

**DOI:** 10.3389/fsurg.2022.865040

**Published:** 2022-05-16

**Authors:** Xing Yan, Changhong Liu

**Affiliations:** Thoracic Surgery Department, The Second Affiliated Hospital of Dalian Medical University Thoracic surgery, DaLian, China

**Keywords:** liquid biopsy, minimal residual disease, lung cancer, non-blood, tumor

## Abstract

Lung cancer is one of the most fatal malignant tumors in the world. Overcoming this disease is difficult due to its late diagnosis and relapse after treatment. Minimal residual disease (MRD) is described as the presence of free circulating tumor cells or other tumor cell derivatives in the biological fluid of patients without any clinical symptoms of cancer and negative imaging examination after the treatment of primary tumors. It has been widely discussed in the medical community as a bridge to solid tumor recurrence. Radiology, serology (carcinoembryonic antigen), and other clinical diagnosis and treatment methods widely used to monitor the progression of disease recurrence have obvious time-limited and -specific defects. Furthermore, as most samples of traditional liquid biopsies come from patients’ blood (including plasma and serum), the low concentration of tumor markers in blood samples limits the ability of these liquid biopsies in the early detection of cancer recurrence. The use of non-blood-derived fluid biopsy in monitoring the status of MRD and further improving the postoperative individualized treatment of patients with lung cancer is gradually ushering in the dawn of hope. This paper reviews the progress of several non-blood-derived fluid samples (urine, saliva, sputum, and pleural effusion) in detecting MRD in lung cancer as well as selecting the accurate treatment for it.

## Background Introduction

Currently, the percentage of patients with lung cancer who die due to its recurrence is very high (within two years, 45% of patients with stage IB and 76% of patients with stage IIIA relapse) (1). However, after decades of related exploration, the methods of lung cancer patient management have gradually undergone profound changes, and it is becoming increasingly clear that the monitoring of disease recurrence is the basis of its successful treatment. The NCCN guidelines for colorectal cancer mention that minimal residual disease (MRD) testing can assess the risk of recurrence of colorectal cancer. Moreover, China has recently reached the first consensus on the detection and clinical application of MRD in lung cancer (2). After early radical resection or late systemic therapy (radiotherapy and chemotherapy) of lung cancer, a small number of cancer cells or their derivatives still remain in the tissue or the body (3). Such minimal residual lesions, which represent the persistence and clinical progression of lung cancer, have been paid increasing attention as the “culprit” of tumor recurrence because they cannot be detected using traditional lung cancer detection methods. At present, liquid biopsy is one of the new methods that is used to detect MRD. Certain biomarkers present in the blood and other body fluids are derived from solid tumors. These biomarkers including circulating tumor cells (CTC), circulating tumor DNA (ctDNA), and extracellular vesicles (EV) can be analyzed using next generation sequencing (NGS) and PCR-based tests and further used for several purposes. For example, ctDNA uses gene sequencing to analyze genetic variations in tumors. It can help in detecting driving gene mutations and drug resistance and longitudinal screening in patients with advanced lung cancer MRD (4). CTC count analysis or ctDNA detection after the resection of cured tumors is helpful for the early monitoring and detection of MRD, and it is also beneficial in initiating adjuvant therapies to prevent recurrence. In addition, the RNA contained in tumor-derived exosomes may also be an independent predictor of the survival of patients with non-small cell lung cancer (NSCLC) (5).We compared the above three biomarkers in Table 1. In contrast to tissue biopsy, liquid biopsy is a non-invasive procedure that can identify tumor-driven mutations, track tumor evolution, and monitor disease recurrence multiple times continuously. Additionally, the mode of operation of liquid biopsy is more convenient, and it can provide the information about the progress of the disease dynamically. It can also help in supplementing the relatively lower quantities of thoracoscopic or bronchoscopic tissue biopsies. It has also been reported that fluid biopsies can prevent approximately 5% of the major complications associated with CT-guided lung biopsies (6). With the advent of the era of precision medicine, the treatment of advanced lung cancer MRD will gradually progress from traditional chemotherapy to molecular targeting and immunotherapy. The detection of tumor-driven genes is the premise of treatment decision-making. However, false-positive results may lead to unnecessary further testing and invasive procedures, whereas false-negative results may delay the necessary treatment. Recently, the concept of fluid biopsy has expanded. Studies have found that the body fluids other than blood (such as urine, saliva, sputum, pleural effusion, and bronchoalveolar lavage fluid [BALF]) may contain more tumor information than blood. At the same time, it can reflect the heterogeneity of tumors in more detail, compared with blood. Thus, non-blood-derived fluid biopsies can provide more opportunities for improving the individualized targeted therapy and prognosis of patients with lung cancer MRD.

**Table 1:** Comparison of the three common biopsy subjects in body fluids.

Biopsies	Source	Advantage	Challenge
CTC	It falls from the primary tumor or metastasis, develops epithelial-stromal transformation, and finally enters the peripheral circulation	High specificity of the tumor cells. Direct quantitative analysis of the separation. Morphological, molecular, and further biological analysis of the tumor cells	The content is rare. Relatively low sensitivity and specificity of isolating tumor cells (technical challenge)
ctDNA	Disposing products derived from dead CTC or active secretion of tumor cells are a small fraction of circulating free DNA (cfDNA) fragments	High sensitivity for detecting DNA alterations (somatic mutations, insertion and deletion, copy number changes, gene fusion)	It is difficult to distinguish between ctDNA and circulating DNA derived from normal cells. There was no functional analysis
Exosome	All cell types release exosomes, but they are abundant in tumor cells, and their main nucleic acids include microRNA (miRNA), tRNA and long non-coding RNA (lncRNA), and a amounts of fragmented mRNA。	Nucleic acids are protected from degradation by dual lipid layers. RNA analysis (miRNA, mRNA, long-coding RNA, RNA-specific variant, RNA expression)	It is difficult to distinguish between tumor-derived exosomes and normal cell-derived exosomes. Extraction difficulty

## Advantages of Non-Blood-Derived Fluid Biopsy

Currently, fluid biopsies from different body fluid sources are used for their application in early postoperative recurrence risk assessment, suitable adjuvant therapy selection, late tumor treatment response tracking, and medication guidance for lung cancer MRD. With the progression, metastasis, and recurrence of solid tumors, a large number of lung cancer cells and their derivatives enter the peripheral blood, making it the most commonly used liquid biopsy material (7, 8). However, all the liquid biopsy-based detection methods face certain problems such as low ctDNA content in plasma, rich cell-free DNA (cfDNA) background produced by normal (non-tumor) cells, and uncertain sources of gene variation (9, 10). With the progress of technology, the sensitivity of personalized gene sequencing technologies in tracking more mutations and thereby detecting low-volume MRD is gradually increasing. However, the clinical application of liquid biopsy is limited due to its operating cost (11). The obvious advantage of some non-blood-derived fluid biopsies is that the exfoliated tumor cells can be collected from the body fluids in direct contact with the tumor, ensuring the content of tumor markers and their correlation with the primary tumor. This can help in reducing the need for complex gene sequencing technologies. Zhang et al. (12) confirmed the advantage of non-blood-derived fluid biopsy by comparing various tumor quantitative indices in tumor tissue, urine, and blood through NGS in 59 patients with pathologically proven bladder cancer. The results reveal that the tumor cell fraction, mutation rate, and mutation load are always consistent between the primary tumor and urine. Further, genetic variations, including EGFR3 changes and ERBB2 amplification, are identified in urine. A considerable proportion of the DNA aberrations in the blood is derived from clonal hematopoiesis. Taking the tumor tissue as a reference, the values of urine DNA detection specificity = 99.3%, sensitivity = 86.7%, positive predictive value = 67.2%, negative predictive value = 99.8%, and diagnostic accuracy = 99.1%, all of which are much better than those of blood. In addition, the study reports that the higher the abundance of utDNA after surgery, the higher is the recurrence rate and the lower is the survival time. EGFR is a membrane receptor that is frequently expressed in tumors (13). Secondary EGFRT790M mutations are the key factor to reduce the effectiveness of tyrosine kinase inhibitor (TKI) treatment (14). As most patients with advanced lung cancer MRD inevitably acquire drug resistance mutations, timely and elongated monitoring of the drug response can prolong the duration of complete remission and thus benefit the patients considerably. Despite of no direct contact with the tumor, the body fluids other than blood not only ensure a certain tumor correlation, but their biopsy also has the advantages of being more painless, non-invasive, and easy to operate. Additionally, non-blood-derived fluids have a large sample size. WU et al. (15) collected 15 patients with lung cancer who were confirmed to have EGFR-sensitized mutations and had received first-line EGFR-TKI treatment. During the course of the treatment, different body fluids were used for the analysis. The results of the longitudinal analysis show that the objective remission rates in tissue, plasma, sputum, and urine are 67%, 67%, 63%, and 66%, respectively. Progression-free survival times of patients with EGFR-sensitized mutations are similar in plasma (7.5 months), sputum (7.9 months), and urine (7.3 months) *p* = 0.721. Su et al. (16) reported that in patients with colorectal cancer, when DNA is extracted from 10 µL of body fluids in each mutation test, the mutated KRAS DNA detection is comparable in serum, plasma, and urine. However, when using a large amount of body fluids (200 µL), the detection rate of the *KRAS* gene in urine (95%) is significantly higher than that in serum (35%) or plasma (40%). Therefore, non-blood-derived fluid biopsy allows longitudinal evaluation and continuous monitoring of tumor occurrence, development, and post-treatment follow-up because of its non-invasive nature, tumor proximity, ease of learning, and economic feasibility. If applied in the dynamic monitoring of lung cancer MRD, it is likely to improve the current situation of the low MRD detection rate, high recurrence, and poor prognosis of lung cancer.Blood is compared to other body fluids in Table 2.

**Table 2:** Comparison of different body fluid samples.

Body fluid type	Main biopsy components	Advantage	Challenge
Blood	CTC, ctDNA, exosome	Mature analysis method. Tumor progression can be monitored in real-time. For the most frequent patients. It is used more commonly and widely used	High false negative rate. Low biomarker quality and quantity. Biomarkers have a shorter half-life. Can’t collect continuously
Urine	CTC,ctDNA, Urine-mRNA, exosome	The sample size is large. Non-invasive, and can be collected continuously. Suitable for longitudinal follow-up. There was a higher response to the tumor metastasis	The collection process is susceptible to contamination. The number was decreased after kidney filtration
Saliva	Saliva-mRNA, miRNA	Non-invasive operation. Low risk of cross-contamination. Lung cancer progression can be monitored in real-time.	Sample size is less. Low biomarker quality and quantity. Complex components, including enzymes, antibodies, hormones, etc
Bronchoalveolar lavage fluid	Tumor cell BALF-mRNA, miRNA, lncRNA, exosome	Non-invasive operation. Early-stage central lung cancer has a high sensitivity and specificity. Intratumoral heterogeneity. The sample size is large	Sample size is less. Lack of mature sampling methods
Pleural effusion	CTC, ctDNA, PE-mRNA	High quality and quantity of the biomarkers. Highly correlated with the clinical characteristics	Invasive operation. Lung cancer progression cannot be monitored in real-time. Early-stage patients are not applicable

## Non-Blood-Derived Fluid Sample Biopsy

### Saliva Biopsy

Saliva is produced by the acinar cells in the salivary glands, which are highly permeable and surrounded by rich capillaries, so that the molecules in the blood can freely exchange with those in the neighboring salivary cells (17–19). Importantly, the saliva is composed of biomolecules, such as proteins, messenger RNA (mRNA), microRNA (miRNA), enzymes, and immunoglobulins from different sources. Analysis of the two kinds of body fluid exocrine proteins reveal that more than 80% of the coincidence rate is shared (20–22), and about 40% of the tumor markers in the blood can also be found in the whole saliva (23). During the development of lung cancer, the salivary glands are stimulated by nerve growth factors released from the primary tumor, resulting in significant changes in the salivary RNA profile of patients with lung cancer. Thus, differentially expressed RNA can be used for the detection of lung cancer (24, 25). In addition, the collection of saliva is fast, simple, cheap, and non-invasive, indicating that it can be regarded as an ideal liquid biopsy specimen. Gu et al. (26) applied the plasma CTC and salivary mRNA biomarkers for the non-invasive detection of NSCLC for the first time. Their sensitivity and specificity are as high as 92.1% and 92.9% respectively, in distinguishing the patients with early lung cancer from the healthy controls. In a study done by Zhang et al. (27), human saliva samples were collected from 42 patients with lung cancer and 74 healthy controls. Saliva transcripts from the patients with lung cancer were analyzed using a gene chip, and the mRNA biomarkers were verified using the real-time fluorescence-based quantitative polymerase chain reaction (RTFQ-PCR). Simultaneously, a new independent cohort of 32 lung cancer samples from 12 patients with lung cancer and 64 matched control samples were independently pre-validated. Seven of the twelve upregulated genes were verified, namely *BRAF*, *CCNI*, *EGFR*, *FGF19*, *FRS2*, *GREB1*, and *LZTS1*. These seven mRNA biomarkers show significant differences between the lung cancer and matched control groups (*p* < 0.05). The area under the curve (AUC) value was 0.707–0.850. The logistic regression model combining the five mRNA biomarkers (*CCNI*, *EGFR*, *FGF19*, *FRS2*, and *GREB1*) can distinguish patients with lung cancer from normal controls (area under the receiver operating characteristic curve [AUC-ROC] = 0.925; sensitivity = 93.75%; specificity = 82.81%). Their expression patterns are related to the occurrence of lung cancer (*p* < 0.05). This study further demonstrates the potential utility of the salivary RNA biomarkers in the lung cancer recurrence detection and risk assessment. Salivary tumor biochemical indicators, such as the imidazole compound (IC) concentration and salivary lactate dehydrogenase (LDH) activity, are also significantly correlated with the survival in the patients with lung cancer (28). The combination of these two parameters is more effective for evaluating the prognosis and survival rate of lung cancer. A study reveals that for the patients with good prognosis (IC < 0.311 mmol/L and LDH > 1,133 U/L), the one, three, and five years survival rates are two times higher than those with poor prognosis. The sensitivity and specificity of the other salivary biochemical indicators such as Cyfra21-1 are 43% and 89%, respectively. The sensitivity and specificity of CEA are 57% and 92%, respectively and those of SCC are 75% and 90%, respectively. The sensitivity and specificity of HCE are 23% and 98% and those of ProGRP are 78% and 95%, respectively (29). These salivary biomarkers have high specificity and can be matched with the tumor genetic tests to reduce false-positive results. This can further be used to check the effectiveness of the treatment, monitor the disease progression, and identify the residual tumors and can play a more accurate role in the development of technology.

### Urine Biopsy

Urine is easier to store and transport. Moreover, it is easier to extract DNA from large samples of urine because it contains reduced levels of some proteins that are present in the blood (such as DNA enzyme). This is because these proteins get filtered by the kidneys, thereby reducing the interference with PCR amplification (30, 31). The possibility of obtaining critical disease information from the urine samples provides more options to complement the traditional tumor sampling methods (32). Chen et al. (33) analyzed 150 urine samples from the patients with NSCLC. The overall coincidence rate of the tissue and urine ctDNA samples is 88% (confidence interval = 0.310–0.700). Immediately after the TKI treatment, a corresponding decrease in the amount of the free DNA is observed, which is unrelated to the *EGFR* mutation in the patient. Notably, they report that the decrease in the free DNA in urine is more significant than that in the tissue by 2.8 times. Li (34) reports that the overall consistency of the urine and blood EGFR spectra of all the patients is 80%, indicating that the sensitivity of the cfDNA obtained from the urine is comparable to that of the plasma. Simultaneously, radiology was used for control analysis and observation. During treatment with gefitinib, there is a significant positive correlation between the tumor size and urine DNA content in the EGFR-positive patients. These results are very helpful in tracking the dynamic changes and mutation status of the cellular free DNA caused by the EGFR-TKI therapy and for further evaluating its correlation with the tumor burden in patients with advanced NSCLC MRD.

The biological basis of urine biopsy is that the cancer cells can release cancer-derived components into the body fluids by secreting exocrine particles, thus protecting them from degradation during circulation and helping them to enter the urine through the filtration of blood by the kidneys (35). The expression profile of the long non-coding RNA (lncRNA) in exocrine bodies is related to the growth, metastasis, and angiogenesis of lung cancer (36). Lin (37) analyzed the differential expression of 640 lncRNAs in the urine exocrine bodies of 20 patients with lung cancer and corresponding healthy individuals by microarray and verified it using quantitative PCR (qPCR). The expression levels of lnc-FRAT1-5, lnc-SRY-11, and lnc-RNASE13-1 are upregulated, whereas those of lnc-RP11-80A15.1.1-2, lnc-ARL6IP6-4, and lnc-DGKQ-1 are downregulated. Further, their expression patterns are related to the occurrence of lung cancer. The results of this study show that these six lncRNAs may regulate the PI3K/Akt, FOXO, or p53 signal pathway. Moreover, they may be an important regulator of the NSCLC, thereby becoming the potential markers of the postoperative MRD metastasis of NSCLC.

Compared with other urinary biomarkers, the metabolic markers are considered to be the proximal markers of disease phenotype, which can specifically observe the changes in early diagnosis and prognosis of patients with lung cancer during the clinical screening. As new cancer metabolic biomarkers, creatine riboside and N-acetylneuraminic acid in the urine have received increasing attention in the targeted metabonomics research in recent years (38). In a case-control study, the National Cancer Institute (39) used a novel ultra-high pressure liquid chromatography-tandem mass spectrometry method to evaluate the urine and blood of more than 1,000 patients of stage I-II NSCLC. The clinical uses of the tumor markers [CA19-9, CA125, CA15-3, and carcinoembryonic antigen (CEA)] have a high false-negative rate. It is also verified that the levels of two metabolites, creatine riboside and N-acetylneuraminic acid, significantly increase in the patients with lung cancer. Compared with the adjacent non-tumor tissues, these two metabolites are present in more quantity in the tumor tissues. They positively correlate with the urine levels, suggesting that they are directly related to the tumor metabolism and can be used as potential non-invasive markers for the prognosis of lung cancer and real-time visualization of the dynamic changes in it.

### Pleural Effusion

Malignant pleural effusion (MPE) usually occurs in the advanced malignant lung tumor cells (40, 41). Currently, the exocrine markers and cfDNA are the two main targets for exploring the progression of lung cancer by the MPE. Therefore, the MPE can be used as an important biomarker source for studying the tumor mutations in the liquid biopsies (42). In addition, compared to the pathological specimens, the rate of gene mutations in patients with lung cancer-related MPE is much higher. If genomic detection can be achieved using more available pleural effusion samples, accurate targeted drug therapy for the patients with advanced NSCLC MRD will be possible, which will have an important clinical and practical value. Additionally, a sufficient source of MPE provides a rich opportunity for the evaluation of tumor genomics (43).

A series of somatic mutations may occur in the lung tumor tissues during the progression of lung cancer. Mutations, such as the EGFRT790M, develop drug resistance and affect the overall survival rate of the patients (44). Dynamic tracking of the tumor-specific mutations using the pleural effusion samples is gradually being verified. In a study by Liu et al. (45), pairs of sufficiently matched tumor tissue samples from 21 patients with lung cancer were compared with MPE cell blocks, MPE supernatant samples, and plasma samples using mutant-specific immunohistochemistry. The sensitivity and specificity of detecting EGFR mutations are (81.8%, 80%, 67.5%) and (71.4%, 100%, 100%), respectively. The consistency of the first two is 81% and that of the latter is 84.9%. Tong et al. (46) performed a study involving 30 patients with lung cancer with pleural effusion and 33 healthy controls. Their results reveal that the total cfDNA concentration (median: 278.1 ng/mL) in the supernatant of PE is much higher than that in the plasma (median: 20.4 ng/mL) (*p* < 0.001). Additionally, 93% of the driving mutations identified in the tumor tissues are detected in the PE-cfDNA, including the changes in ALK, BRAF, EGFR, ERBB2, KRAS, NF1, PIK3CA, and RET, whereas only 62% of those are detected in the plasma cfDNA. The higher mutation allele frequency is 98.4% in the PE-cfDNA, 90.5% in the PE-sDNA, and 87% in the plasma cfDNA, indicating that the PE-cfDNA is more representative of the tumor heterogeneity than the PE-sDNA or plasma cfDNA. This further implies that the PE-cfDNA is an independent source of the tumor DNA and therefore less affected by the abundance of tumor cells. The tumor mutation burden (TMB), an independent biomarker of immunotherapy response, of the PE-cfDNA is similar to that of the tumor tissue but significantly higher than that of the PE-sDNA (median TMB: 3.3) and plasma cfDNA (median TMB: 3.4). Thus, as a target substitute for the traditional fluid biopsy, the PE-cfDNA also provides a higher accuracy for identifying patients with an immunotherapy response.

The circulating tumor extracellular RNA is considered as a minimally invasive biomarker for the cancer diagnosis. However, it appears in the form of short fragments and originates from the apoptotic cells, which complicates the molecular analysis (47). In contrast, nucleic acids present in the exocrine bodies of body fluids have the advantage of being protected from any degradation by the double lipid layer, thereby providing them more stability (48). Many exocrine markers in blood are used in the clinical diagnosis and treatment of lung cancer. The changes in the exocrine bodies in pleural effusion can also be used as an alternative method for lung cancer biopsy. Song et al. (49) used targeted NGS of 416 cancer-relevant genes. The results of the study reveal that 304 gene mutations are detected in 18 cfDNA samples, and 276 gene mutations are detected in 20 exoDNA samples. In 18 patients with both exoDNA and cfDNA samples, 47 mutations are detected in 8 genes (*EGFR*, *ALK*, *KARS*, *BRAF*, *MET*, *PTEN*, *TP53*, and *RB1*). Of the 47 mutations, 43 are common between the two types of samples, with a coincidence rate of 89.6%. Tamiya et al. (50) analyzed and verified small RNA (miRNA) biomarkers in the pleural effusion samples of 56 patients with lung cancer. The results reveal that four candidate miRNAs (miR-21, miR-31, miR-182, and miR-210) are identified, and their expression patterns are related to the occurrence of lung cancer. Among them, miR-182 and miR-210 are verified in independent queues using RT-PCR, and they are increasingly upregulated. The AUC values for distinguishing the benign and malignant effusions are 0.87 (sensitivity = 92.7%; specificity = 73.3%) and 0.81 (sensitivity = 58.5%; specificity = 93.3%). The joint evaluation of miR-182 and miR-210 results in an AUC value of 0.88 (95% confidence interval = 0.78–0.97). These results highlight the use of exocrine miRNAs in improving the performance of the differential diagnosis of pleural effusion and in compensating for the lack of prediction of the therapeutic effect of MRD for lung cancer. In addition, exocrine body lncRNAs exist in the pleural effusions, but their potential as a tumor biomarker has never been deeply studied in the MPEs caused by lung cancer. Berta et al. (51) analyzed the EV-related miRNA maps of 25 cases of pleural effusion using the TaqMan OpenArray technique. The results show that miRNA-1-3p, miRNA-144-5p, and miRNA-150-5p are the best diagnostic markers. The accuracy of these markers in the diagnosis of lung cancer is 0.941, 0.882, and 0.912, respectively. Wang et al. (52) discussed the clinical significance of lncRNA in pleural effusion. The results reveal that three types of lncRNAs (MALAT1, H19, and CUDR) can be used as the diagnostic indices of lung cancer-MPE. The sensitivity and accuracy of the combined prediction of these lncRNAs verified by the logistic model are 84.8% and 90.9% (AUC, 0.924), respectively, which are higher than those of CEA (88%, AUC, 0.826). Concurrently, other studies have evaluated the diagnostic value of biomarkers such as CYFRA21-1, CEA, CA19-9, CA15-3, and CA125 in the pleural effusion and found that the accuracy of these biomarkers is relatively low (40.5%–85.3%) (53). The above studies also confirm, for the first time, the feasibility of the combination of CEA and lncRNA biomarkers present in pleural effusion in the early risk assessment of lung cancer. In addition, the expression of the baseline MALAT1 in the pleural effusion of patients with lung cancer-MPE is negatively correlated with the efficacy of chemotherapy. Thus, it has the potential to monitor the therapeutic effects as well as improve the prognosis of the patients with MRD lung cancer during adjuvant therapy.

### Bronchoalveolar Lavage Fluid

BALF is a clinical examination in which normal saline is injected into the alveoli using bronchoscopy and then aspirated under negative pressure to obtain the alveolar cells and biochemical components (39). The BALF is anatomically in direct contact with the tumor site, thus having a higher quality and quantity of biomarkers than the other biological fluid samples and thereby being more accurate for the monitoring changes in lung cancer (55, 56). Hur et al. (57) report a new liquid biopsy method for EGFR genotyping using BALF EV DNA which significantly increases the detection rate of mutations compared with the traditional EGFR genotyping method. Identification of the EGFR mutations is the first step in the treatment (EGFR-TKIs and cytotoxic chemotherapy) of patients with early and newly diagnosed advanced NSCLC. All the cases of *EGFR* mutations can be detected in the tumor tissues (*n* = 31) along with six additional mutations. The compliance rates for the first, second, third, and fourth stages are 79%, 100%, 74%, and 92%, respectively. With the progression of TNM staging, especially when there is metastasis, the coincidence rate increases significantly (*p* < 0.05). The sensitivity and specificity are 75.9% (95% confidence interval = 62.1%–86.1%) and 86.7% (95% confidence interval = 77.1%–92.9%), respectively. Sensitive detection of the EGFR mutations can provide more effective and tolerant treatment with the EGFR-TKIs in patients with advanced MRD.

DNA methylation is the key to regulating gene expressions and maintaining the cell characteristics. Epigenetic changes in the DNA methylation play an important role in the occurrence of lung cancer and can be used for its early detection (58). Zhang et al. (59) report that the methylation level in BALF is closely related to that in the lung cancer biopsy samples. Among the candidate genes, *SHOX2* and *RASSF1A* are the most sensitive molecular markers for the early detection of lung cancer. Using RT-PCR and Sanger sequencing, the sensitivity and specificity of the SHOX2 and RASSF1A methylation in BALF are 81.0% and 97.4%, respectively (95% confidence interval = 0.849–0.943). Compared with the established cytological examination (sensitivity = 68.3%; specificity = 97.4%; AUC value = 0.828; 95% confidence interval = 0.777–0.880) and serum biomarker CEA (sensitivity = 30.6%; specificity = 100.0%; AUC value = 0.741; 95% confidence interval = 0.670–0.812), the SHOX2 and RASSF1A methylation groups show the highest diagnostic efficiency. Notably, the combination of cytology and SHOX2 and RASSF1A methylation analysis can significantly improve the diagnostic efficiency as well as the status identification of MRD in lung cancer. Additionally, the SHOX2 and RASSF1A in BALF show significant methylation differences before and after the surgical treatment of early lung cancer (*p* < 0.0001). Thus, they can be used for the early diagnosis of recurrence after MRD treatment.

Currently, the peripheral blood CTC count is the most recognized fluid biopsy method for monitoring the recurrence of MRD, but its clinical application is limited due to the inadequate number of CTCs and absence of well-developed separation technologies (60). CTCs need to undergo a series of complex processes, such as exfoliation from the primary tumor or metastatic focus and epithelial-mesenchymal transformation, before they finally get into the patient’s peripheral circulation (61). In contrast, the lavage fluid goes directly to the tumor site, which makes it easier to detect the tumor cells isolated from BALF than from the peripheral blood. Therefore, theoretically, the diagnostic performance of tumor cell detection in the BALF would be better than that in the CTCs during the early stage of lung cancer, as the number of tumor cells is related to poor prognosis and shorter progression. Zhong et al. (62) reveal that the detection rate of the CTCs in the peripheral blood of patients with lung cancer is 90.5% (218/241), which is similar to that of the tumor cells in BALF (46/51, 90.2%). At the same sample size, the number of tumor cells detected in patients with lung cancer in the BALF is significantly higher than those in the CTCs in the peripheral blood (6.76 ± 0.89 vs. 5.78 ± 0.57) *p* = 0.016. The diagnostic performance of the tumor cells in BALF for relapse after treatment (sensitivity = 0.829; specificity = 0.869; AUC = 0.871; ROC = 0.963) is also significantly higher than that of the serum tumor markers, such as NSE, CEA, and CA125 (all AUC-ROC < 0. 6, all *p* < 0. 05). The reason may be that serum tumor markers are metabolites, which are usually produced and released by tumor cells. Surprisingly, there is almost no difference between the CTCs and tumor tissues in BALF detection (AUC-ROC = 0.858 and 0.850, respectively). With further optimization of the BALF detection technology, the use of BALF tumor cells to supplement or even replace the peripheral blood CTC detection method will serve the MRD patients with lung cancer more accurately.

## Summary and Prospect

Compared with traditional fluid biopsy, the emerging non-blood-derived fluid biopsy lacks the consensus and clinical verification of the pre-treatment and analytical methods. The body fluids collected from certain organs or parts of the body cannot reflect the specific conditions of cancer metastasis in the same manner as the blood circulating throughout the body (63). For example, the urine contains many components in it that may not have been filtered by the kidneys. However, these components may be absent in the blood. Moreover, it contains the cells that fall off from the genitourinary system. Therefore, urine biopsy has been further studied mostly in the genitourinary system cancers and is considered as one of the main limitations of the other non-urinary system cancers. In addition, compared with the blood, the other body fluids have a more complex microbial environment, and their microflora is dynamic, varying with changes in the local and systemic conditions. Microorganisms and their metabolites can have unpredictable impacts on the MRD biopsy results. In some body fluids, the low abundance of tumor genes (DNA, mRNA, etc.), lack of stable targeted molecules, and risk of contamination during sample preparation may lead to false-positive results and overdiagnosis (64). In terms of methodology, different analytical methods include NGS, RT-PCR, and microarray. Advances in the sequencing technology have enabled the detection of mutations and chromosome changes related to tumor biology. However, there is still a lack of large-scale multicenter studies on the non-blood-derived fluid samples, which needs to make a tradeoff between the sensitivity, specificity, availability, cost, and practicality. This is also the main reason that hinders its application in clinical practice.

Biomarkers derived from liquid samples are urgently required to monitor the MRD, predict the tumor response, and explore the drug resistance in the clinic. Non-blood-derived fluid biopsy, as an alternative method for analyzing genetic variations, provides a non-invasive method to detect the changes in lung cancer in advance. Furthermore, it also complements the results of traditional blood biopsy tests. Therefore, more patients with cancer can receive an accurate treatment. However, there are still many problems to be solved pertaining to MRD itself. Future research will be necessary to determine how the information from tumor biopsy, clinical examination, and medical imaging can be combined with the genomic and MRD information from liquid biopsies in the best way. Finally, in this era of individualized and accurate medical treatment, the application of multiple liquid biopsy samples in the clinical oncology will become a new way to guide the clinical decision-making and also improve the patient prognosis. Moreover, with the development of related research, it is believed that non-blood-derived fluid biopsy will become an important part of routine screening and clinical management of lung cancer and other malignant tumors.
